# Towards effective screening for paternal perinatal mental illness: a meta-review of instruments and research gaps

**DOI:** 10.3389/fpubh.2024.1393729

**Published:** 2024-06-25

**Authors:** Philipp Schöch, Laura Hölzle, Astrid Lampe, Christine Hörtnagl, Ingrid Zechmeister-Koss, Anna Buchheim, Jean Lillian Paul

**Affiliations:** ^1^Department of Psychiatry, Psychotherapy, Psychosomatics, and Medical Psychology, Division of Psychiatry I, Medical University Innsbruck, Innsbruck, Austria; ^2^Department of Clinical Psychology II, Institute of Psychology, University Innsbruck, Innsbruck, Austria; ^3^Ludwig Boltzmann Institute for Rehabilitation Research, Vienna, Austria; ^4^Austrian Institute for Health Technology Assessment, Vienna, Austria

**Keywords:** paternal perinatal depression, paternal mental health, perinatal, depression, anxiety, fathers, screening instruments

## Abstract

**Background:**

Paternal perinatal mental illness (PPMI), which affects around one in 10 fathers, is under-recognised despite increasing awareness of men’s mental health in the perinatal period. Social stigma and men’s reluctance to seek help exacerbate this gap. Neglecting the mental health needs of new fathers not only puts them at increased risk for mental illness themselves, but also has a profound and long-lasting impact on their families, children and their own self-esteem as they navigate their new role in the family dynamic.

**Objective:**

This meta-review systematically identifies instruments assessing PPMI symptoms, evaluates their psychometric properties and applicability, presents key findings from studies using these tools, and identifies gaps and limitations in the literature on PPMI symptom assessment.

**Methods:**

A systematic literature review was conducted using search strategies applied to PubMed, PsycNet APA, Cochrane, and Web of Science, supplemented by hand searches. Relevant information was extracted from each included study. Extracted data were analysed narratively to address the research questions.

**Results:**

Findings identified limitations and gaps in current screening practices. While the Edinburgh Postnatal Depression Scale (EPDS) is the most widely used screening tool for both fathers and mothers, it inadequately captures atypical depressive symptoms in men. Cutoff scores lack consensus, and instrument sensitivity varies significantly due to cultural and sociodemographic factors. A number of other screening tools have been identified, most of which are more general and not specifically designed for perinatal mental health.

**Conclusion:**

This meta-review broadens perspectives on PPMI screening instruments, highlighting key themes, patterns, and differences across the included reviews. While a variety of screening tools are used, the review underscores the necessity for tools specifically tailored to fathers during the perinatal period.

## 1 Introduction

In recent years, there has been a growing acknowledgment of the significance of paternal perinatal mental illness (PPMI) within perinatal research (1). This emerging research emphasizes the critical need for early identification and support for fathers experiencing psychological disorders during their partner’s pregnancy to 1 year postpartum. There is also a growing acknowledgment of the profound impact the transition to parenthood has on paternal mental health and overall family well-being (2). Fatherhood has undergone a significant transformation, particularly in western societies, from rigid, traditional gender roles to a more flexible framework characterised by negotiation and adaptation of parental responsibilities within families (3). While acknowledging the diversity of fathering experiences, it is clear that cultural norms influence parenting roles differently across regions. For example, in countries such as Sweden, active parenting, including shared parental leave and childcare involvement, is typical, while in countries such as Spain, traditional gender roles often persist (4). Active fatherhood is influenced by institutional frameworks such as parental leave policies, cultural norms and values, company culture, as well as individual and partnership factors (5). In Austria, fathers are increasingly acknowledged as crucial participants in family care. However, despite positive shifts in attitudes, their actual involvement remains limited, with only 17% taking parental leave. Traditional gender roles and societal norms serve as significant barriers, highlighting the need for stronger policy measures to encourage a more equitable sharing of family responsibilities (6). Nevertheless, there is a discernible trend across contemporary Western societies where fathers are actively challenging traditional societal expectations. This trend is characterized by a more equitable sharing of housework, childcare, and work responsibilities (7). This societal shift reflects evolving norms and the dynamic nature of family structures. As fathers strive to balance the demands of work and family life, they are prioritising meaningful engagement with their children, contributing to a more equitable distribution of parental roles. This increased involvement highlights the importance of paternal mental health, particularly during the perinatal period, as fathers take on more active parenting roles.

PPMI is associated with an increased risk of inter-parental conflict, higher relationship dissatisfaction, and potential difficulties in infant temperament, highlighting the broader negative impact on family functioning (8). When fathers are emotionally unavailable, it can create tensions within the family dynamics and which may lead to emotional neglect in children, impacting children’s self-esteem, emotional regulation, and social skills development. Additionally, children may develop insecure attachment styles and struggle to form trusting relationships as adults. Emotionally unavailable fathers may inadvertently model unhealthy emotional behaviours or reinforce gender stereotypes, affecting children’s understanding of gender roles (9).

A meta-analysis by Cameron et al. (10) estimated the prevalence of paternal depression during their partner’s pregnancy and up to 1 year postpartum to be around 8%. Depression rates seemed to be higher in the third to sixth months postpartum period and lower during the second trimester of pregnancy. It is important to note that these estimates only include depression and not other types of mental health problems during this period. Other studies that examined diagnosed mental health problems and above-threshold symptoms found that approximately 5–10% of fathers had perinatal depression, and approximately 5–15% experienced perinatal anxiety (10–12).

Still, Fisher et al. (2) suggests that PPMI may be underestimated because men are less likely to report traditional symptoms of depression and may express depression differently for example, by engaging in harmful coping behaviours, such as aggression, substance use, and suicide. Using traditional measures to screen for depression in fathers may give an inaccurate picture of their mental health. Psouni et al. (13) also highlighted a considerable variation in the reported prevalence of paternal postpartum depression (PPPD), which they attribute to a lack of uniform assessment for PPPD, lack of consensus regarding the time period to be considered, or uncertainty about whether minor depression, as defined by DSM-IV (14), should be included.

The study of Psouni et al. (13) underscores the prevalence of “depressive equivalents” in fathers’ symptoms, serving as counterparts to traditional indicators of depression. This implies that an assessment tool, incorporating both typical depression and externalizing (depressive equivalent) symptoms may be more appropriate for evaluating paternal depression. Strikingly, Psouni et al. (13) showed that 83% of fathers scoring above the BDI-II (Beck Depression Inventory II) cut-off for suspected depression had not disclosed their condition to a healthcare professional.

Identifying and addressing PPMI is complicated by various barriers to help-seeking that have been identified, including misconceptions and lack of knowledge about paternal perinatal depression (PPPD), adherence to masculine norms, and the stigma surrounding PPPD (15, 16). Findings from Pedersen et al. (16) interview study indicate that adherence to masculine ideals and parental inequality within the family and healthcare system hinder fathers’ help-seeking behavior. They argue that feelings of being the secondary parent could question the father’s legitimacy for his own mental healthcare needs. Psouni et al. (13) revealed that a significant percentage of fathers experiencing depressive symptoms avoid seeking professional help. That is consistent with the observed low help-seeking behavior commonly observed in men with depression (17).

### 1.1 Objectives and research questions

Evidence suggests that fathers may manifest depressive symptoms differently to mothers, necessitating an exploration of available screening tools to better capture PPMI. The primary aim of this paper is to analyze existing reviews of screening tools to assess paternal mental health during their partner’s pregnancy and the first year postpartum, and to examine the instruments used. It seeks evidence on the reliability and validity of these instruments, while identifying limitations, potential biases and research gaps in the literature on PPMI assessment.

## 2 Methods

To address the research objective, a systematic review was conducted, adhering to the PRISMA guidelines (18). Given the significant heterogeneity in study designs and outcomes among the included reviews on screening tools for PPMI, a meta-review as described by Hunt et al. (19) and using a narrative synthesis approach was selected and considered appropriate. We used narrative synthesis (20) to qualitatively summarize and compare the findings and characteristics of the reviews without attempting to combine their results quantitatively.

### 2.1 Literature search

The following databases were searched for relevant articles: PubMed, PsycNet APA, Cochrane, and Web of Science. The search was conducted across all time periods until August 30, 2023, to ensure the inclusion of the recent and relevant studies. A systematic literature search was conducted using the following search strategy, with a combination of keywords and phrases related to PPMI and screening tools:

((“paternal” OR “father*” OR “dad”) AND (“perinatal mental health” OR “postpartum depression” OR “post-partum”)) AND ((“screen*” OR “identif*” OR “specific measure”) AND (“depression” OR “anxiety” OR “stress” OR “well-being”)) AND (“systematic” OR “meta-analysis” OR “review”).

### 2.2 Study selection and eligibility criteria

After screening retrieved articles at the title and abstract level for relevance, those deemed potentially eligible were selected for full-text review (PS). Subsequently, a second reviewer (LH) independently reviewed the identified articles to verify their eligibility for inclusion. Utilizing the eligibility criteria outlined below, both primary reviewer (PS) and second reviewer (LH) independently assessed the full-text articles for inclusion. Any discrepancies or uncertainties regarding study inclusion were resolved through consensus discussion between PS and LH to ensure adherence to the eligibility criteria.

### 2.3 Inclusion and exclusion criteria

Studies were included if they met the following criteria:

- Reviewed screening tools for fathers and/or partners during the perinatal period.
- Were published as systematic reviews, meta-analyses, or reviews.

Studies were excluded if they:

- Were not written in English.
- Were conference abstracts or non-peer-reviewed publications.
- Did not specifically focus on PPMI screening.
- Did not provide sufficient information on the screening tools assessed.

### 2.4 Data extraction and synthesis

Data were systematically extracted from included reviews to provide a comprehensive overview on the assessment of PPMI. Data exctracted included: author names, publication years, study countries, number of primary studies, research focus, key results, instrument-specific findings, and included instruments. To synthesize the extracted data, we used a narrative approach was employed, focusing on identifying patterns and gaps in the literature regarding screening practices for PPMI. This synthesis aimed to distill the collective insights from the included reviews and to provide a narrative on the current research landscape of PPMI screening.

### 2.5 Quality assessment

The quality assessment of the included systematic reviews, meta-analyses, and reviews was conducted by both primary reviewer (PS) and second reviewer (LH) using the AMSTAR 2 Tool (21). This tool was selected for its capability to enhance the transparency and reliability of the quality assessment process. Both PS and LH independently assessed the methodological quality of each included study using the AMSTAR 2 criteria, and any discrepancies or uncertainties were resolved through consensus discussion to ensure consistency and accuracy in the assessment.

### 2.6 Synthesis

A narrative synthesis (20) was chosen as it is versatile, suitable for various review questions, and for its ability to synthesize and interpret findings from multiple studies, particularly when a statistical meta-analysis may not be feasible. To organize the findings from the included reviews, we utilized tables to outline their specific characteristics, facilitating the identification of patterns. In structuring our analysis, we opted to identify overarching themes and center the description around these emergent themes, rather than adhering strictly to predefined data categories. We believe this approach provides a comprehensive understanding of the literature and that synthesizing and interpreting findings allows to generate insights beyond the mere aggregation of data points, thus enriching the overall narrative.

## 3 Results

**Figure 1:**
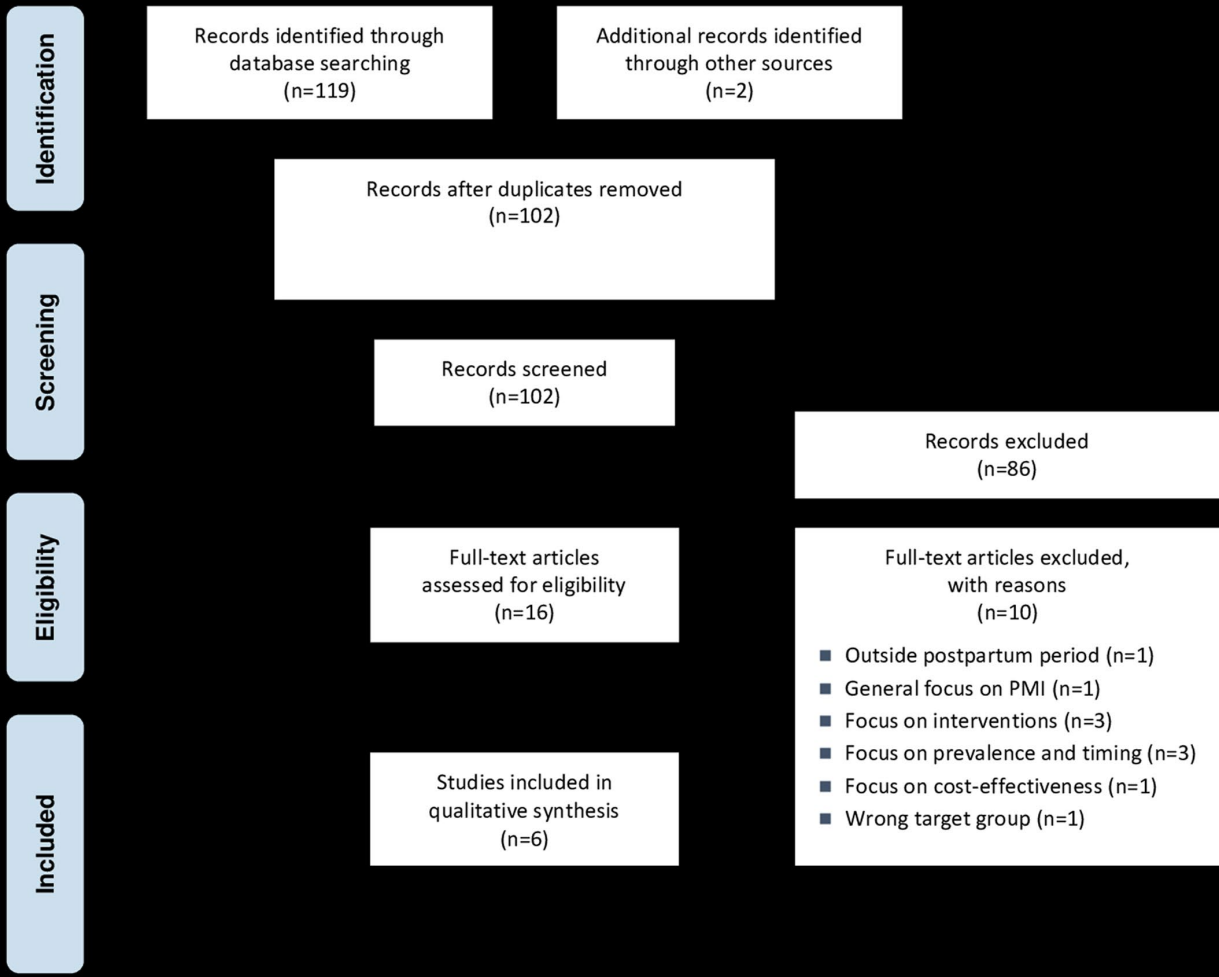
Modified PRISMA flow diagram based on Page et al. (18)

The electronic searches identified 119 records (see Figure 1). In addition, two further records were identified through a supplementary search, resulting in 102 records after removing duplicates. Screening at the title/abstract level resulted in 16 records being obtained in full, with six studies ultimately included in the review.

### 3.1 Characteristics of included reviews

A total of six reviews (1, 4, 22–25) were included in this meta-review (see Table 1). These were published between 2015 and 2022. They collectively examined 167 primary studies published from 1987 to 2021 and indicating a surge in research over the last decade. Although studies have been conducted around the world, many were concentrated in Europe (75), the United States (23), and Australia (17). Within Europe, there was a notable concentration of research from the United Kingdom (15), Sweden (12) and Italy (10). The scope of the reviews primarily focused on screening tools for paternal mental health during both the prenatal and postnatal periods, with one review additionally considering other co-parents and partners.

**Table 1:** Characteristics of included reviews.

Review	Author	Year	Countries	Primary studies	Research focus	Key results	Instrument-specific findings	Included instruments
1	Berg et al.	2022	USA (14) Australia (4) Canada (2 China (3 England (2) Finland (2) Italy (7) Japan (3) Portugal (3) Sweden (2) Taiwan (2) Turkey (2) Other (13)	59	Instruments used to identify symptoms of PPPD and their characteristics and measurement properties	13 instruments used to measure PPPD symptoms during pregnancy and postpartum in 25 countries Only 6 of 13 analyzed instruments have been subject to validation for PPPD EPDS is the most extensively assessed and validated instrument for measuring PPPD, followed by the CES-D and BDI EPDS is more accurate than other instruments (GMDS, PAPA, and PHQ) in detecting PPPD None of the instruments were specifically developed to measure symptoms of PPPD The lack of gender-specific items in EPDS may lead to under-detection of PPPD symptoms and it is unclear whether EPDS and the other instruments uniquely identify depressive symptoms or a broader state of mind characterized by distress and anxiety	The EPDS was examined most extensively, with 38 studies reporting on its measurement properties. It demonstrated moderate to high internal consistency and validity for identifying depression in postnatal fathers EPDS has good internal consistency with a Cronbach’s alpha above 0.70 in 34 of the 38 studies reporting on it, while across all instruments, internal consistency ranged from 0.60 to 0.91 Cutoff scores used to detect depression varied across studies, with the EPDS having optimum cutoff scores from 5 to 13 The BDI was examined in four studies, reporting good internal consistency and validity for detecting PPPD The GMDS was examined in two studies, demonstrating fair to moderate reliability and moderate correlation with EPDS The K10 and K6 were examined in one study, showing good internal consistency and weak correlations with scales completed by partners The PAPA was examined in one study, showing high internal consistency and significant associations with EPDS The PHQ-9 was examined in two studies, showing good internal consistency and validity but less accuracy compared to EPDS for detecting PPPD	Beck Depression Inventory (BDI) Brief Symptom Inventory (BSI) Center for Epidemiologic Studies Depression Scale (CES-D) Chinese Health Questionnaire (CHQ) Depression Anxiety and Stress Scale (DASS) Edinburgh Postnatal Depression Scale (EPDS) Gotland Male Depression Scale (GMDS) Hospital Anxiety and Depression Scale (HADS) Kessler Psychological Distress Scale (K10) Paternal Adjustment and Paternal Attitudes Questionnaire (PAPA) Patient Health Questionnaire (PHQ) Postpartum Depression Screening Scale (PDSS) Zung’s Self-rating Depression Scale (SDS)
2	Kennedy and Munyan	2021	Australia (1) China (1) Italy (1) Japan (1) Saudi Arabia (1) Sweden (3) UK (1) Vietnam (1)	10	Scientific evidence regarding the sensitivity of screening measures for PPPD	All studies included the Edinburgh Postnatal Depression Scale (EPDS) Significant variability in cut-off scores used and sensitivity and specificity among populations Wide variation in the prevalence of depression among fathers in the postpartum period across studies. Estimates of the prevalence ranged from 2.5 to 28.3% across studies Due to low help-seeking behavior among men, improved sensitivity of depression screening tools is needed for the prevention and treatment of PPPD symptoms Lower levels of education and socioeconomic status and other demographic factors increase PPPD risk Neither the EPDS nor the GMDS may be adequate for screening, and a combination of scales may be necessary Cultural variations in the presentation of depressive symptoms among fathers. Different cultural contexts may influence the interpretation and reporting of symptoms Cultural variations in recommended cut-off scores	The EPDS is sensitive to symptoms of depression and distress but may be less sensitive to depression itself EPDS: Different studies have used different cut-off scores (ranging from 7 to 12 or more), and its sensitivity and specificity have varied in different populations GMDS: Focuses on typical male depressive symptoms such as aggression and irritability. It may be more sensitive in detecting distress as it includes items related to irritability and external reactivity BDI: One study reported a sensitivity of 100% and a specificity of 81% for the BDI in detecting depression in fathers HAD: Recommended cut-off scores for the HAD anxiety subscale varied (from 4 to 8), and sensitivity ranged from 23.3 to 51% Other instruments (e.g., PHQ-9, CES-D): Sensitivity and specificity varied for these instruments, suggesting differences in their performance in different populations and contexts	Beck Depression Inventory-II (BDI-II) Center for Epidemiological Studies Depression Scale (CES-D) Edinburgh Gotland Depression Scale (EGDS) Edinburgh Postnatal Depression Scale (EPDS) General Health Questionnaire 12 items (GHQ-12) Gotland Male Depression Scale (GMDS) Hospital Anxiety and Depression Scale (HADS) Hospital Anxiety and Depression Scale—Anxiety subscale (HADS-A) Patient Health Questionnaire (PHQ-9) Structured Clinical Interview for DSM-IV Axis II
3	Pérez et al.	2017	Europe (30) Asia (10) USA (6) Australia (4) Brazil (2)	52	Identification and description how PPPD and/or depressive symptoms in men have been assessed during the first year of fatherhood	A variety of instruments were employed, with the Edinburgh Postnatal Depression Scale (EPDS) being the most common. Other tools included clinical interviews, the Beck Depression Inventory (BDI), the Hospital Anxiety and Depression Scale (HADS), among others A total of 20 instruments were identified across studies 39 studies reported the mean age of the fathers as 32.7 years (SD = 5.83), with a range between 27 and 36 years The prevalence of PPPD symptoms was highest in Sweden (47%) and lowest in Turkey (1.8%) The prevalence of PPPD symptoms ranged from 1.8 to 47%, with a mean prevalence of 11.9% which was attributed to differences in study designs, populations, and cultural factors across the included studies The time of assessment varied across studies, with some studies assessing depressive symptoms during pregnancy, and others assessing them postpartum The prevalence of PPPD symptoms varied across different cut-off scores on the EPDS or other scales used in the study	EPDS was applied in almost every study (*N* = 40), and in most of them (*N* = 25) it was the only screening tool administered Hospital Anxiety and Depression Scale (HADS): This scale was used in five studies to assess depressive symptoms Beck Depression Inventory (BDI-I): The BDI-I was used in three studies Patient Health Questionnaire Depression Module (PHQ-9): was used in two studies Center for Epidemiologic Studies (CES-D): was used in four studies Birmingham Interview for Maternal Mental Health, Fifth Edition (BIMMH): this instrument was used in one study General Health Questionnaire (GHQ-12): was used in two studies Mental Health Index (MHI-5): was used in one study Positive and Negative Affect (PANAS): was used in one study Semi-Structured Clinical Interview (SADS-L): was used in one study Mini Neuropsychiatric Interview (MINI): was used in one study Blues Questionnaire: was used in two studies Zung’s Self-rated Anxiety Scale (Zung SAS): was used in one study	EPDS (Edinburgh Postnatal Depression Scale) CES-D (Center for Epidemiologic Studies-Depression Scale) GHQ-12 (General Health Questionnaire) BDI (Beck Depression Inventory) SCID (Structured Clinical Interview) SADS-L (Schedule for Affective Disorders and Schizophrenia-Lifetime) HADS (Hospital Anxiety and Depression Scale) MINI (Mini Neuropsychiatric Interview) PHQ-9 (Patient Health Questionnaire Depression Module) MHI-5 (Mental Health Index) PANAS (Positive and Negative Affect) Zung SAS (Zung’s Self-rated Anxiety Scale) BELA (self-designed questionnaire on feelings of stress) SF-36 (36-Item Short Form Health Survey-Taiwanese version) BIMMH (Birmingham Interview for Maternal Mental Health, fifth edition) Blues Questionnaire VAS (Visual Analog Scale) Brief PHQ SCL-90-R (Symptom Checklist 90-Revised) EPDS-P (Edinburgh Postnatal Depression Scale—partner version)
4	Shafian et al.	2022	Portugal (1) Australia (1) UK (1) Hong Kong (1) Vietnam (1) Sweden (1) Saudi Arabia (1)	7	Identification of suitable cut-off scores for the use of EPDS in the screening for PPPD by collating data available from EPDS validation studies	Various diagnostic instruments were used across studies, including Structured Clinical Interview for DSM-IV (SCID), Primary Care Evaluation of Mental Disorders (PRIME-MD), Schedule for Affective Disorders (SADS), Diagnostic Interview Schedule (DIS), and Diagnostic and Statistical Manual of Mental Disorders, Fifth Edition (DSM 5) Prevalence of PPPD varied widely across studies (2.9 to 23.8%) Mean age of the study participants varied from 26.2 to 35 years old Factors affecting estimates of PPPD prevalence include screening tool used, diagnostic criteria, and timing of assessment Clinicians may consider using lower cut-off scores ranging from 7 to 10 of EPDS screening for PPPD as it confers the optimum balance between sensitivity and specificity	Positive likelihood ratio increased with higher EPDS cut-off scores, ranging from 3.31 to 13.16 Sensitivity and specificity of the EPDS varied across studies, with sensitivity ranging from 40 to 100% and specificity from 58.1 to 93% for different cut-off scores. The pooled sensitivity decreased with increasing cut-off points, while specificity increased Fixed-effect meta-regression showed that the accuracy of EPDS did not vary significantly according to depression prevalence, mean age of fathers, translation of EPDS, or country of origin of the research	Edinburgh Postnatal Depression Scale (EPDS) 12-item General Health Questionnaire (GHQ-12) Zung’s Self-rated Anxiety Scale (Zung SAS) Hospital Anxiety and Depression Scale (HAD-A) Beck Depression Inventory (BDI) Patient Health Questionnaire-9 (PHQ-9) Centre for Epidemiologic Studies Depression Scale (CES-D) Schedule for Affective Disorders (SADS) Structured Clinical Interview for DSM-IV (SCID) Primary Care Evaluation of Mental Disorders (PRIME-MD) Diagnostic Interview Schedule (DIS) Diagnostic and Statistical Manual of Mental Disorders, Fifth Edition (DSM 5)
5	Darwin et al.	2021	UK (10) Italy (2) USA (3) Sweden (4) Australia (5) Portugal (1) Hong Kong (1) Vietnam (1)	27	Identification and synthetization of evidence on the performance of mental health screening tools and the acceptability of mental health assessment, specifically in relation to fathers, other co-parents and partners in the perinatal period	EPDS is the most commonly used tool across studies No consensus on the appropriate cut-point for identifying depression or anxiety in fathers using the EPDS Despite the variability in cut-points, most studies found that the EPDS performed similarly or better than other assessment tools when used for fathers Cultural variations in emotional expression influence prevalence and could influence the choice of cut-points Stigma around mental health and the perception of traditional gender norms are assessment barriers Professionals lack training and confidence in addressing PPMI Identified challenges regarding acceptability of PPMI screening were categorized at the individual-, practitioner- and service-level	The EPDS has been used to assess both depression and anxiety, with some studies suggesting its use for screening fathers, while others caution against its routine use due to concerns about false positives and poor sensitivity Various studies recommend different thresholds for EPDS, ranging from ≥5 to ≥13 for differentiating between distress and non-distress	Edinburgh Postnatal Depression Scale (EPDS) Beck Depression Inventory (BDI) Patient Health Questionnaire (PHQ-9) General Health Questionnaire (GHQ) Hospital Anxiety and Depression Scale (HADS-A) Zung’s Self-rated Anxiety Scale (SAS) Structured Clinical Interview for DSM-IV (SCID) Schedule for Affective Disorders (regular and lifetime versions) Psychiatric Assessment Scale Diagnostic Interview Schedule Primary Care Evaluation of Mental Disorders (Prime-MD)
6	Edward et al.	2015	UK (1) Australia (2) Norway (1) Brazil (2) Sweden (2) China (2) Japan (1) Spain (1)	12	Research on PPPD and identifying potential screening and referral options	Maternal depression has been identified as the strongest predictor of PPMI Other risk factors for paternal PPPD include a personal history of depression, high prenatal symptom scores for depression and anxiety, an unsupportive relationship, unemployment, financial/life stressors, etc. PND in fathers can negatively affect both the father-child relationship and the relationship between parents. It can lead to increased parenting stress, lower bonding with the infant, and higher risk of emotional and behavioral problems in children While primarily intended for mothers, some studies have applied the EPDS to fathers Different studies had different cutoff values, highlighting the variability in using the EPDS as a screening tool for depression in men Studies indicate prevalence rates of paternal PND ranging from 3.4 to 14% Routine screening and assessment of both parents should occur across the pregnancy and postnatal period Detailed assessment of fathers during the postnatal period, especially when their female partners are depressed	Studies have found different optimal EPDS cutoff scores for fathers compared to mothers due to differential response to certain items, such as the “crying” item. For fathers, a cutoff of 5/6 was found to be optimum for identifying distress (depression or anxiety disorders) EPDS does not diagnose depression but can indicate the need for further assessment EPDS identified 5% of fathers with depressive symptoms using a cutoff of >10, compared to 3.4% with a male depression scale (GMDS)	Edinburgh Postnatal Depression Scale (EPDS) Beck Depression Inventory (BDI) Center for Epidemiologic Studies Depression Scale (CES-D) General Health Questionnaire (GHQ) General Health Questionnaire-28 (GHQ-28) State Anxiety Inventory (SAI) Postpartum Bonding Questionnaire (PBQ)

The systematic data extraction and narrative analysis conducted as part of this meta-review identified a diverse range of screening tools utilized in the assessment of PPMI (see Table 2). The analysis involved a detailed examination of various dimensions extracted from included reviews, including study countries, number of primary studies, research focus, key results, instrument-specific findings, and included instruments. This analysis highlights details about the instruments utilized, their characteristics, and measurement properties, providing specific findings regarding the performance of these instruments in detecting symptoms of PPMI. The analysis revealed notable variations in the use and efficacy of different screening instruments across studies. Furthermore, the analysis identified limitations of currently used screening tools and areas demanding further investigation to enhance screening practices for PPMI.

**Table 2:** Overview of screening instruments mentioned in included reviews.

Screening instrument	Type of screening	Number of items	Description
Edinburgh Postnatal Depression Scale (EPDS)	Perinatal Mental Health	10	Screens for postpartum depression in mothers
Birmingham Interview for Maternal Mental Health (BIMMH)	Perinatal Mental Health	None	Assesses maternal mental health during the perinatal period
Edinburgh Postnatal Depression Scale, partner version (EPDS-P)	Perinatal Mental Health	10	Screens for postpartum depression in partners (fathers)
Postpartum Bonding Questionnaire (PBQ)	Perinatal Mental Health	25	Developed to provide an indication of mother–infant relationship problems
Paternal Adjustment and Paternal Attitudes Questionnaire (PAPA)	Perinatal Mental Health	30	Assesses paternal adjustment and attitudes
Blues Questionnaire	Perinatal Mental Health	10–20	Screening for postpartum depression in mothers
Beck Depression Inventory (BDI)	Depression	21	Measures the severity of depressive symptoms
Beck Depression Inventory-II (BDI-II)	Depression	21	Assesses the presence and severity of depressive symptoms
Brief Symptom Inventory (BSI)	Depression	53	Evaluates various psychological symptoms, including depression
Center for Epidemiologic Studies Depression Scale (CES-D)	Depression	20	Screens for depressive symptoms in the general population
Depression Anxiety and Stress Scale (DASS)	Depression	42	Measures the emotional states of depression, anxiety, and stress
Gotland Male Depression Scale (GMDS)	Depression	13	Designed to assess depression specifically in males
Kessler Psychological Distress Scale (K10)	Depression	10	Measures psychological distress, including depression
Patient Health Questionnaire (PHQ)	Depression	9	Screens for common mental disorders, including depression
Postpartum Depression Screening (PDSS)	Depression	35	Specifically designed for screening postpartum depression
Self-rating Depression Scale (SDS)	Depression	20	Self-report measure of depressive symptom
Edinburgh Gotland Depression Scale (EGDS)	Depression	12	Designed for assessing depression in males
Hospital Anxiety and Depression Scale (HADS)	Anxiety/Depression	14	Screens for anxiety and depression in hospital patients
Hospital Anxiety and Depression Scale-Anxiety subscale (HADS-A)	Anxiety	7	Specifically assesses anxiety symptoms in hospital patients
Zung’s Self-rated Anxiety Scale (SAS)	Anxiety	20	Measures the severity of anxiety symptoms
State Anxiety Inventory (STAI-X1)	Anxiety	20	Assesses state anxiety (current, momentary anxiety)
HAD-A (Hospital and Anxiety Depression Scale)	Anxiety	7	Screens for anxiety symptoms in hospital patients
HADS-ASAS (Hospital Anxiety and Depression Scale-Anxiety and Stress subscales)	Anxiety	14	Assesses anxiety and stress in hospital patients
Positive and Negative Affect (PANAS)	Other Mental Health	20	One of the most widely used scales to measure mood or emotion
General Health Questionnaire–28 (GHQ-28)	Other Mental Health	28	Screens for general mental health and distress
Chinese Health Questionnaire (CHQ)	Other Mental Health	12	Assessment tool of psychological distress
Visual Analog Scale (VAS)	Other Mental Health	None	Measuring subjective experiences such as pain or mood
Primary Care Evaluation of Mental Disorders (PRIME-MD)	Other Mental Health	None	Screening and diagnosing common mental disorders in primary care
General Health Questionnaire 12 items (GHQ-12)	General Mental Health	12	Screens for general mental health and distress
International Classification of Diseases (ICD-8/10)	General Mental Health	None	A standardized diagnostic classification system for mental disorders
Mental Health index (MHI-5)	General Mental Health	5	Brief assessment of mental health in adults
Mini Neuropsychiatric Interview (MINI)	General Mental Health	None	A short, structured diagnostic interview for mental disorders
Schedule for Affective Disorders and Schizophrenia (SADS)	General Mental Health	None	A comprehensive diagnostic interview for mood disorders
Structured Clinical Interview for DSM-IV Axis II Personality Disorders (SCID)	General Mental Health	None	Semi-structured interview guide for making diagnoses
Symptom Checklist 90-Revised (SCL-90-R)	General Mental Health	90	Assesses a wide range of psychological symptoms
36-Item Short Form Health Survey, Taiwanese version (SF-36)	General Mental Health	36	Measures health-related quality of life
Chinese Health Questionnaire (CHQ)	General Mental Health	30	Screening for mental health issues in Chinese populations

The AMSTAR 2 assessment revealed mixed methodological rigour among the reviewed studies. While some showed strengths in protocol clarity, comprehensive searches, and detailed study descriptions, others were lacking in areas such as protocol transparency, duplicate data extraction, and bias assessment (see Tables 3, 4). Notably, Shafian et al. (25) demonstrated robust methodology, including rigorous statistical analysis, likely due to its inclusion of a quantitative meta-analysis.

**Table 3:** AMSTAR 2 assessment.

Items	Berg et al. (1)	Kennedy and Munyan (24)	Pérez et al. (4)	Shafian et al. (25)	Darwin et al. (22)	Edward et al. (23)
1. Did the research questions and inclusion criteria for the review include the components of PICO?	+	+	+/−	+/−	+	+
2. Did the report of the review contain an explicit statement that the review methods were established prior to the conduct of the review and did the report justify any significant deviations from the protocol?	+/−	−	−	−	−	−
3. Did the review authors explain their selection of the study designs for inclusion in the review?	+	−	−	+	+	−
4. Did the review authors use a comprehensive literature search strategy?	+	+	+	+	+	+
5. Did the review authors perform study selection in duplicate?	+	+	+/−	+	+	+/−
6. Did the review authors perform data extraction in duplicate?	+	+	+/−	+	+	+/−
7. Did the review authors provide a list of excluded studies and justify the exclusions?	−	−	−	−	+	−
8. Did the review authors describe the included studies in adequate detail?	+	+	+	+	+	+
9. Did the review authors use a satisfactory technique for assessing the risk of bias (RoB) in individual studies that were included in the review?	−	−	−	+	+	+
10. Did the review authors report on the sources of funding for the studies included in the review?	−	−	−	−	−	−
11. If meta-analysis was performed did the review authors use appropriate methods for statistical combination of results?	N.a.	N.a.	N.a.	+	N.a.	N.a.
12. If meta-analysis was performed, did the review authors assess the potential impact of RoB in individual studies on the results of the meta-analysis or other evidence synthesis?	N.a.	N.a.	N.a.	+	N.a.	N.a.
13. Did the review authors account for RoB in individual studies when interpreting/ discussing the results of the review?	N.a.	N.a.	N.a.	+	N.a.	N.a.
14. Did the review authors provide a satisfactory explanation for, and discussion of, any heterogeneity observed in the results of the review?	−	−	−	−	−	−
15. If they performed quantitative synthesis did the review authors carry out an adequate investigation of publication bias (small study bias) and discuss its likely impact on the results of the review?	N.a.	N.a.	N.a.	+	N.a.	N.a.
16. Did the review authors report any potential sources of conflict of interest, including any funding they received for conducting the review?	+	+	−	+	+	+

**Table 4:** Additional information on AMSTAR 2 quality assessment of included reviews.

Review	Author	Year	Quality of review	Evidence base for review
1	Berg et al.	2022	+ Scoping review in accordance with the five-stage methodological framework proposed by Arksey and O’Malley + Comprehensive search strategy (PRISMA-ScR Checklist), scoured multiple databases and explored grey literature sources + Cronbach Alpha to evaluate the reliability of studies measuring PPPD + Pairs of reviewers selected studies based on predetermined criteria and one reviewer extracted the data while another checked its accuracy. The two resolved differences through re-examination of the publication and discussion + Extracted data included measurement properties such as internal consistency, reliability, measurement error, content validity, construct validity, and responsiveness. + Measurement properties are defined based on the COSMIN framework + PICO components were not explicitly mentioned, but it addresses similar components +/− There's no explicit mention of justifying deviations from the protocol − No mention of risk of bias assessment of the included studies − There's no discussion of heterogeneity in the paper	Peer reviewed studies Cross-sectional: 21 studies (35.6%) Longitudinal: 26 studies (44.1%) Validation: 12 studies (20.3%)
2	Kennedy and Munyan	2021	+ Review provides an a priori design by clearly stating its purpose + Authors used a structured approach outlined by Whitmore and Knafl (29) for integrative review construction, instead of PICO + Clear methodology to guide the literature search process, including problem identification, literature search, data evaluation, data analysis, and results presentation + Inclusion and exclusion criteria were well defined, and the search strategy was comprehensive + Clear summary of the included studies in a table format − The authors did not explicitly explain their selection of study designs for inclusion in the review − No mention of risk of bias assessment of the included studies − Did not provide a list of excluded studies, and did not assess the quality of the included studies − There's no discussion of heterogeneity in the paper	Articles reporting on the psychometric properties of the screening measure used to detect PPD in men, published in English, and peer-reviewed
3	Pérez et al.	2017	+ Search strategy was comprehensive, as they included multiple databases and used keywords to search title or abstract + Well defined inclusion and exclusion criteria + Data extraction is specified + Multiple reviewers were involved in the review process +/− It is unclear if the authors performed study selection and data extraction in duplicate +/− Unclear if the components of PICO or similar were used − Research questions not mentioned − No risk of bias assessment of the included studies − There's no discussion of heterogeneity in the paper	Peer-reviewed studies documenting depression or depressive symptoms in men within the first trimester to one-year postpartum
4	Shafian et al.	2022	+ PRISMA guidelines were followed + Study protocol was registered with PROSPERO + Literature search was conducted on multiple databases, with a broad search strategy + Selection criteria were clearly defined, and the data extraction and analysis were conducted systematically + Use of QUADAS-2 tool to assess the quality of selected studies +/− Unclear if the components of PICO were explicitly mentioned in the registration or protocol	Peer-reviewed studies that compared EPDS scores for depression with validated diagnostic interviews
5	Darwin et al.	2021	+ Clear design and objectives + Systematic and rigorous methods + Comprehensive literature search and citation chaining + Study selection process involved a team of reviewers + Inclusion and exclusion criteria were defined +Quality appraisal to assess study strengths and weaknesses, adhering to systematic review guidelines + Quality appraisal of included studies with CASP and QUADAS-2 − There’s no discussion of heterogeneity in the paper	Peer reviewed studies. Accuracy of screening tools in the included studies was determined by comparison of screening tool with diagnostic interview. Acceptability of screening tools was predominantly assessed through parents’ and health professionals’ perspectives
6	Edward et al.	2015	+ Systematically addressed PPD by defining primary and secondary outcomes + Inclusion and exclusion criteria are well-defined + Comprehensive search strategy across multiple databases, and applying specific inclusion and exclusion criteria + Quality appraisal was conducted with CASP and Cohort Studies methodological checklist + Data extraction done by two researchers + Process of data extraction and synthesis was transparently described +/− It is unclear if the authors performed study selection and data extraction in duplicate − No specific details about the synthesis methods used − There's no discussion of heterogeneity in the paper	Peer-reviewed studies including qualitative and quantitative research, longitudinal studies and cross-sectional studies

### 3.2 Frequently used assessment tools in the context of PPMI

The Edinburgh Postnatal Depression Scale (EPDS) has been analysed in nearly every single study of the included reviews, and can be considered the primary screening tool for evaluating PPMI and associated mental health concerns in fathers or partners throughout the perinatal phase (see Table 1). The EPDS is a 10-item self-report instrument that takes approximately 5 min to complete and is designed to assess mood and emotional wellbeing in the postpartum period. Scores range from 0 to 30, with 10 or more points suggesting the possibility of depression of varying severity (26). Three other widely used instruments for assessing the mental health of new fathers are: Beck Depression Inventory (BDI), Center for Epidemiologic Studies Depression Scale (CES-D), and Patient Health Questionnaire (PHQ). The BDI (27), although not tailored for the perinatal period, comprises 21 questions designed to assess the severity of depressive symptoms. In contrast, the CES-D (28), which encompasses 20 questions, serves as a comprehensive self-reported questionnaire for assessing depressive symptoms in community populations. It covers cognitive, somatic, and psychological aspects and is also valuable for screening new fathers. The PHQ, including its various versions such as the PHQ-9 (30), is a widely used screening tool for assessing depression severity. The PHQ-9 consists of nine questions specifically designed to evaluate depressive symptoms experienced over the past 2 weeks. While the BDI, CES-D, and PHQ-9 are not explicitly designed for perinatal mental health, the included reviews showed that they can be useful to screen for depression symptoms in fathers or partners during the perinatal period.

### 3.3 Sensitivity and specificity challenges

While various screening tools have been examined, few were specifically designed to detect postpartum depression (PPPD), with only two targeting male symptomatology: the Perinatal Assessment of Paternal Affectivity (PAPA) and the Gotland Male Depression Scale (GMDS). The GMDS has demonstrated effectiveness comparable to the EPDS in identifying PPPD using a cutoff score of ≥13. A Danish study described the reliability of the GMDS as fair to moderate, with a Cohen’s kappa (*κ*) value of 0.49, indicating some agreement with the EPDS in identifying PPPD. Additionally, a Swedish study found a moderate correlation between the GMDS and the EPDS (*r* = 0.76) (1). However, research suggests that neither the GMDS nor the EPDS alone are sufficient for screening males in the postpartum period (24). Data on the sensitivity and specificity of the PAPA tool are limited, as only one study reviewed by Berg et al. (1) analyzed its performance. This study, conducted in Portugal, reported high internal consistency for the PAPA (0.91 antenatal, 0.90 postnatal) and significant correlation with the EPDS, with cutoffs of ≥95 for PAPA-AN and ≥92 for PAPA-PN.

The EPDS is the most extensively studied and validated tool for assessing postpartum depression in both men and women. In Berg et al. (1) review, they found that the EPDS exhibited internal consistency, with a Cronbach’s alpha exceeding 0.70 in 34 out of 38 studies. While an alpha above 0.70 is typically considered acceptable, it is important to consider the context of the EPDS’s 10-item scale, as this may influence the interpretation of these findings. Across all other instruments considered in Berg’s review, internal consistency values ranged from 0.60 to 0.91. Cutoff scores used to detect depression varied across their studies, with the EPDS having optimum cutoff scores from 5 to 13 for fathers. Despite variations in cutoff points, most studies indicated that the EPDS performed as well as or better than other assessment tools when used for fathers. In Shafian’s et al. (25) review, they recommended focusing on the determination of the appropriate cut-off score for EPDS, and that clinicians should use a lower score between 7 to 10, because it appears to strike a balance between sensitivity and specificity when screening for PPPD.

Despite those findings and suggestions, the determination of an ideal cutoff point for the EPDS, as well as for various other available screening instruments, remains a matter of debate in the reviewed studies. Kennedy and Munyan (24) observed cultural variations in recommended cutoff scores for depression screening instruments, and highlighted the importance of considering context-specific factors. It is important to mention that the absence of gender-specific items in the EPDS may lead to under-detection of symptoms in fathers. Berg et al. (1) addressed the lack of gender-specific items in screening tools and concluded that it is unclear whether they uniquely identify symptoms of depression or a broader state of mind, including distress and anxiety. However, Edward et al. (23) noted that the EDPS is a suitable screening tool that could alert a clinician to the need for a full diagnostic interview.

### 3.4 Symptoms, indicators and identification of PPMI

Kennedy and Munyan (24) highlighted that modern fathers experience increased expectations and responsibilities during the 3 to 4-month postpartum period, including childcare, housework, which is changing their previous social role, while also being perceived as the main income providers. Balancing family life and work demands can lead to distress more often than depression. This distress is characterized by feelings of being overwhelmed, helpless, anxious, irritable, self-blaming, using avoidant/escapist activities (e.g., sports, overworking, excessive time on internet/TV, gambling, substance use), and aggressiveness that may be under detected by traditional depression screenings. The analyzed reviews suggest that the EPDS is sensitive to symptoms of depression and distress, but may be less sensitive to depression itself, especially in fathers. The reviews included in this study show the evident value of using multiple screening tools to assess PPPD to enhance sensitivity and specificity. It also indicates the likeliness that neither scale alone is sufficient for depression screening in new fathers. In this context, Psouni et al. (13) showed that a modified EPDS with GMDS items had greater sensitivity than the EPDS alone.

Another study by Tran et al. (31) highlighted that, while all measures had acceptable reliability, the sensitivity of the EPDS in men was significantly lower than in women. Reviewed studies suggest using lower EPDS cut-off scores for PPPD compared to maternal depression. In this context, the crying item of the EPDS is suggested as one item which could for example lead to underreporting due to gendered differences. Societal expectations and gender differences in emotional expression may lead fathers to underreport or express their distress differently than mothers, making this item less reliable for fathers. Shafian et al. (25) noted that their reviewed studies recommend a lower EPDS cutoff score for fathers in comparison to mothers. However, they stated that the result should be interpreted with caution due to the influence of diverse factors such as culture, socioeconomic status, education, and societal context. Moreover, they stressed that the timing of EPDS administration and variations in how male depression is expressed across different cultures can impact EPDS scores.

### 3.5 Factors contributing to the variation in prevalence of PPMI

This meta-review identified significant variation in the reported prevalence rates of PPMI across the included studies. Pérez et al. (4) conducted a review encompassing 52 single studies and identified prevalence rates based on applied screening tools ranging from as low as 1.8% to as high as 47%, with a mean prevalence of 11.9%. Other included reviews showed similar results, and based on their findings, this wide variation in prevalence can be associated with the screening tool used, diagnostic criteria, and timing of assessment. In regards to the EPDS, sensitivity and specificity showed significant variation across studies for different cutoff scores, with sensitivity ranging from 40 to 100% and specificity from 47.8 to 100%, as demonstrated by Shafian et al. (25). Additionally, the timing of assessments varied across studies, with some focusing on depressive symptoms during partner’s pregnancy and others during the postpartum period. Pérez et al. (4) for example showed in their review that prevalence in Turkey is the lowest and is much higher in Sweden. This is an important observation, as cultural norms and expectations shape how fathers perceive and report their feelings.

### 3.6 Risk factors and barriers to assessment

Kennedy and Munyan (24) highlighted a number of demographic risk factors. They emphasized that lower education levels, low socioeconomic status, a family with three or more children, and single or widowed marital status were significant contributors to PPPD vulnerability. Additionally, a history of psychiatric treatment, unintended pregnancy, and unstable employment situations heightened the risk. In addition to that, Darwin et al. (22) reported multifaceted obstacles to the acceptability of PPMI screening. These challenges were identified across individual, practitioner, and service levels. Among the key issues were gendered perceptions around fatherhood, practitioner knowledge and confidence, time constraints, and the necessity for resources such as tools, training, and referral pathways. Some fathers in their reviewed studies hesitated to seek help due to cultural and social stigma, while others saw potential in routine screening to destigmatize perceptions and discussions around PPMI. Some fathers reported that they would only open up about their mental health if they felt the health visit focused on them as well as their partner. They also mentioned their reluctance to speak in front of their partners and service limitations (especially conflicting service hours with work commitments) as issues. Darwin’s et al. (22) study also noted gaps in professional training and the unequal perception of fathers as caregivers by some child health nurses. Service-level shortcomings, such as an exclusive focus on birthing mothers, hindered fathers’ engagement. Traditional gender role beliefs explain why some fathers in Darwin et al. (22) review felt it was culturally and socially unacceptable to discuss difficulties with fatherhood. In contrast to that, countries with more egalitarian gender models are likely to report higher instances of PPPD (4). This perception of stigma around mental health and needing to be strong are barriers to assessment, especially for those who strongly internalized those traditional gender roles. These insights underscore the complex interplay of societal norms and individual experiences in the realm of PPMI.

### 3.7 Identification of gaps in screening and support

There is a lack of validated and reliable tools for specifically identifying and supporting PPMI during pregnancy and the postpartum period. Reviews show inconsistent use of scales beyond the EPDS and limited analysis of demographic factors impacting sensitivity. Limited research has been conducted on the development of screening tools tailored to new fathers. Pérez et al. (4) advocates moving beyond the EPDS, despite its predominant use in numerous studies, as results have been often inconclusive regarding sensitivity. Their results suggest the development and validation of specialized instruments for screening and diagnosing PPMI. This is based on the observation that males often express depression through atypical (male-specific) symptoms, such as aggression and irritability, rather than exhibiting a typical depressive mood.

## 4 Discussion

A significant increase in studies related to this topic reflects the growing recognition of the importance of including fathers in research within this field. Despite this progress, challenges remain in effectively addressing PPMI. The predominant use of the EPDS highlights the reliance on a tool not explicitly tailored for fathers, raising concerns about its sensitivity and specificity in this population. The EPDS and other screening tools often overlook unique manifestations of PPMI in fathers. The spectrum of applied screening instruments primarily includes either specialized tools tailored to identify symptoms typically associated with maternal depression, such as excessive sadness or mood fluctuations, or non-specialized screening measures for general mental health conditions without a specific gender focus. This issue is also reflected in recent publications (32, 33) highlighting the necessity of gender-specific screening tools for PPMI, pointing out differences in symptom presentation and comorbidities compared to maternal perinatal depression, and advocate for tailored instruments. Men experiencing PPMI may exhibit externalized symptoms like irritability, anger, or increased alcohol consumption, and these are often overlooked. Based on Shafian’s et al. (25) findings, using the EPDS may yield a notable number of false positives, necessitating additional assessments, which can lead to substantial costs for service providers. However, alternative scales used have shown inconsistency across studies and have received limited attention in PPMI research. Comparing the EPDS to these tools (e.g., BDI or PHQ-9), the EPDS has specific questions tailored to postpartum experiences, such as questions about feelings of sadness, guilt or changes in appetite and sleep patterns that are particularly relevant to mothers. Conversely, instruments like the PHQ-9 and BDI encompass a broader spectrum of depressive symptoms that may be encountered by both mothers and fathers. The outcomes of this review underscore that EPDS and GMDS scales measure distinct aspects of paternal depression, supporting the necessity for a more comprehensive approach to its identification and support. Kennedy and Munyan (24) highlighted that neither the EPDS nor the GMDS alone may be adequate for screening, suggesting a combination of scales may be necessary.

Moreover, the variation in prevalence rates reflects the impact of traditional gender roles within different cultures, thereby influencing how fathers respond to questionnaires concerning their emotional wellbeing. Addis (34) emphasized that adherence to traditional gender norms can hinder help-seeking behavior and increase the risk of depression. An additional consideration should be, as Pérez et al. (4) hypothesized, a link between international rates of PPPD and traditional gender role beliefs in their review. They propose that countries with strong patriarchal values, where men are expected to uphold patriarchal ideals (e.g., exhibit dominance and aggression), may have lower rates of identified PPPD. Societal norms may discourage men from expressing their emotions, which can constrict their emotional life and, as a consequence, reduce their susceptibility to PPPD. This idea aligns with previous research, such as Psouni et al. (13) highlighting the coexistence of traditional depressive symptoms and depressive equivalents in fathers, and suggests that a screening tool combining both symptom types may be more appropriate for evaluating PPMI. A study from Salokangos et al. (35) underscores nuanced disparities in how depressive symptoms are reported by men and women. These differences arise from the inclusion of gender-related assessment items, resulting in potentially biased outcomes when measuring depression. Consequently, future research should address the development of assessment tools that mitigate such biases, aiming to enhance diagnostic accuracy and reduce misclassification. However, with respect to cultural differences and expectations, it is also possible that fathers in cultures with strong patriarchal values do not experience PPPD to the same extent as fathers in other cultures. The role of fathers in family dynamics and responsibilities varies greatly across different societies. In cultures where fathers are expected to take on significant caregiving responsibilities and actively participate in childcare, the transition to parenthood might affect their mental health differently compared to cultures where their involvement is minimal. Other factors, such as cultural differences in the understanding and acceptance of mental health issues, variations in healthcare systems, and access to mental health services, could also influence PPPD rates. Consequently, future research should focus on developing assessment tools that account for these complexities, thereby emphasizing the need for culturally sensitive screening approaches tailored to diverse populations.

### 4.1 Recommendation and implications

This paper emphasizes the critical need for valid and culturally sensitive screening tools to detect and support PPMI. Kennedy and Munyan (24) stress the importance of validation studies, particularly considering cultural and socio-demographic variations in cutoff scores. Gressier et al. (36), highlight the link between PPMI and parent–child separation during maternal psychiatric episodes, underlining the importance of involving fathers in screening.

A broader research effort is required to further validate these screening tools and improve our understanding of PPMI. Improved sensitivity in screening instruments is crucial for prevention and treatment. Recommendations include developing combined scale questionnaires and utilizing multiple screening tools, with an emphasis on culturally sensitive approaches (4).

The risk factors associated with PPMI should also be considered when developing screening tools for fathers. A comprehensive meta-analysis by Goodman (37) identified maternal depression as the most robust predictor of paternal postpartum depression. Philpott et al. (38) identified several additional risk factors, previous depression, infants sleep problems, perceived lack of social support, challenging economic circumstances, and no access to or not utilizing paternity leave. Ansari et al. (39) expanded this collection spectrum to include relationship dissatisfaction, low paternal education level, unemployment, work-related stress, low parenting self-efficacy, and perceived stress.

A key challenge identified is the limited involvement of fathers in integrated service provision. While universal screening for fathers is recommended, guidance on optimal timing is still lacking, as noted by Kennedy and Munyan (24). Nevertheless, universal screening could potentially address the issue that Edward et al. (23) mentioned that partners of individuals with PPMI encountered challenges, including a lack of awareness about where to find PPMI resources and difficulties in seeking social support or referrals to healthcare professionals.

In terms of medical encounters and service provision, it would be beneficial to widen the dyadic mother-infant perspective and include the father or partner in the integrated delivery of services. Hambidge et al. (40) emphasize that while fathers may attend some antenatal visits and are typically present at birth, these interactions primarily center around the wellbeing of the mother and baby. Consequently, fathers may perceive their role as secondary to that of the mother and may hesitate to express their need for support. Fletcher et al. (41) highlight fathers’ limited engagement with health services as a significant barrier to their assessment and support during early parenthood. Pérez et al. (4) suggest assessing the co-parental system and emphasize that this assessment should start before birth, with follow-ups during the entire first year postpartum or a long-term perspective on this phenomenon. This could be useful for designing future intervention programs. Moreover, Pedersen et al. (16) suggest that screening for PPMI may facilitate fathers’ help-seeking behavior, requiring further investigation. Research gaps could inform public awareness campaigns, PPMI healthcare guidelines, and healthcare professional training.

Darwin et al. (22) highlighted the ethical challenge of conducting routine PPMI assessments in the absence of follow-up pathways and support services. Identifying PPMI is crucial, but without adequate support, there can be a risk of creating harm for fathers. Establishing accessible interventions alongside screening is essential for ethical and effective PPMI identification. Research can address this gap by informing the development of follow-up pathways and support services.

### 4.2 Strengths and limitations

This study utilizes narrative synthesis to analyze the diverse range of study designs and outcomes among reviewed articles, providing a qualitative and comprehensive analysis. However, the varied study designs posed challenges for certain components of the AMSTAR 2 assessment. It is important to note limitations in applying some elements of the AMSTAR 2 tool to evaluate the methodological quality of systematic reviews in this context. Despite this, the systematic literature search, robust search strategy, and predefined data extraction format enhance the study’s consistency. Additionally, the study’s exclusive focus on reviews may have overlooked relevant individual studies, potentially limiting the current understanding of the topic. Excluding non-English publications may introduce bias by missing valuable literature. The narrative synthesis method does not facilitate quantitative result synthesis, limiting precise statistical conclusions and introducing subjectivity in interpretation.

## 5 Conclusion

Fathers, as with mothers, undergo significant emotional and psychological adjustments during the transition to parenthood. The sleepless nights, the added financial responsibilities, and the emotional rollercoaster of caring for a newborn, can exert an immense toll on fathers’ mental wellbeing. Yet, depression in fathers often remains underdiagnosed and undertreated. Enhancing the sensitivity of mental health screening instruments tailored to fathers is not only a matter of equity, but also one of profound consequence. By fine-tuning these tools to include a wider range of depressive symptoms, it may be possible to identify struggling fathers earlier in their journey, and offer them the support and resources they need to cope effectively. Early detection can be pivotal in preventing the escalation of depressive symptoms and, in turn, reducing the risk of adverse outcomes for fathers, their partner and their children.

## Data availability statement

The original contributions presented in the study are included in the article/supplementary material, further inquiries can be directed to the corresponding author.

## Author contributions

PS: Writing – original draft, Writing – review & editing. LH: Writing – review & editing. AL: Writing – review & editing. CH: Writing – review & editing. IZ-K: Writing – review & editing. AB: Writing – review & editing. JP: Writing – review & editing.

## References

[1] Berg RC Solberg BL Glavin K Olsvold N. Instruments to identify symptoms of paternal depression during pregnancy and the first postpartum year: a systematic scoping review. Am J Mens Health. (2022) 16. doi: 10.1177/15579883221114984 PMC9490477 36124356

[2] Fisher SD Cobo J Figueiredo B Fletcher R Garfield CF Hanley J . Expanding the international conversation with fathers’ mental health: toward an era of inclusion in perinatal research and practice. Arch Womens Ment Health. (2021) 24:841–8. doi: 10.1007/s00737-021-01171-y, PMID: 34431009

[3] Tiedje LB Darling-Fisher C. Fatherhood reconsidered: a critical review. Res Nurs Health. (1996) 19:471–84. doi: 10.1002/(SICI)1098-240X(199612)19:6<471::AID-NUR3>3.0.CO;2-L, PMID: 8948401

[4] Pérez F Brahm P Riquelme S Rivera C Jaramillo K Eickhorst A. Paternal post-partum depression: how has it been assessed? A literature review. Mental Health Prev. (2017) 7:28–36. doi: 10.1016/j.mhp.2017.07.001

[5] Baierl A. Buchebner-Ferstl S. Dörfler-Bolt S. (2023). Vatersein in Österreich Eine empirische Untersuchung im multi-methoden Design

[6] Rille-Pfeiffer C Kapella O. Evaluierung des neuen Kinderbetreuungsgeldkontos und der Familienzeit: Meta-Analyse. Wien: Social Science Open Access Repository (SSOAR) (2022).

[7] Diniz E Brandão T Monteiro L Veríssimo M. Father involvement during early childhood: a systematic review of the literature. J Fam Theory Rev. (2021) 13:77–99. doi: 10.1111/jftr.12410

[8] Ramchandani PG Psychogiou L Vlachos H Iles J Sethna V Netsi E . Paternal depression: an examination of its links with father, child and family functioning in the postnatal period. Depress Anxiety. (2011) 28:471–7. doi: 10.1002/da.20814, PMID: 21506206 PMC3128925

[9] LeFrançois BA. Distressed fathers and their children: a review of the literature. Int J Soc Psychiatry. (2012) 58:123–30. doi: 10.1177/0020764010387478 21106602

[10] Cameron EE Sedov ID Tomfohr-Madsen LM. Prevalence of paternal depression in pregnancy and the postpartum: an updated meta-analysis. J Affect Disord. (2016) 206:189–203. doi: 10.1016/j.jad.2016.07.044 27475890

[11] Leach LS Poyser C Cooklin AR Giallo R. Prevalence and course of anxiety disorders (and symptom levels) in men across the perinatal period: a systematic review. J Affect Disord. (2016) 190:675–86. doi: 10.1016/j.jad.2015.09.063 26590515

[12] Paulson JF Bazemore SD. Prenatal and postpartum depression in fathers and its association with maternal depression a meta-analysis. JAMA. (2010) 303:1961–9. doi: 10.1001/jama.2010.605 20483973

[13] Psouni E Agebjörn J Linder H. Symptoms of depression in Swedish fathers in the postnatal period and development of a screening tool. Scand J Psychol. (2017) 58:485–96. doi: 10.1111/sjop.12396, PMID: 29052228

[14] American Psychiatric Association. Quick reference to the diagnostic criteria from DSM-IV-TR (p. 370). Washington, DC: APA. (2000)

[15] Möller-Leimkühler AM. Barriers to help-seeking by men: a review of sociocultural and clinical literature with particular reference to depression. J Affect Disord. (2002) 71:1–9. doi: 10.1016/S0165-0327(01)00379-2, PMID: 12167495

[16] Pedersen SC Maindal HT Ryom K. “I wanted to be there as a father, but I couldn’t”: a qualitative study of fathers’ experiences of postpartum depression and their help-seeking behavior. Am J Mens Health. (2021) 15:155798832110243. doi: 10.1177/15579883211024375 PMC8202277 34116610

[17] Schuppan KM Roberts R Powrie R. Paternal perinatal mental health: at-risk fathers’ perceptions of help-seeking and screening. The Journal of Men’s Studies. (2019) 27:307–28.

[18] Page MJ McKenzie JE Bossuyt PM Boutron I Hoffmann TC Mulrow CD . The PRISMA 2020 statement: an updated guideline for reporting systematic reviews. Int J Surg. (2021) 88:105906. doi: 10.1016/j.ijsu.2021.105906, PMID: 33789826

[19] Hunt H Pollock A Campbell P Estcourt L Brunton G. An introduction to overviews of reviews: planning a relevant research question and objective for an overview. Syst Rev. (2018) 7:39. doi: 10.1186/s13643-018-0695-8, PMID: 29490699 PMC5831229

[20] Popay J Roberts H Sowden A Petticrew M Arai L Rodgers M . Guidance on the conduct of narrative synthesis in systematic reviews a product from the ESRC methods programme Peninsula Medical School Universities of Exeter and Plymouth (2006).

[21] Shea BJ Reeves BC Wells G Thuku M Hamel C Moran J . AMSTAR 2: a critical appraisal tool for systematic reviews that include randomised or non-randomised studies of healthcare interventions, or both. BMJ. (2017) 358:j4008. doi: 10.1136/bmj.j4008, PMID: 28935701 PMC5833365

[22] Darwin Z Domoney J Iles J Bristow F Siew J Sethna V. Assessing the mental health of fathers, other co-parents, and partners in the perinatal period: mixed methods evidence synthesis. Front Psychiatry. (2021) 11:585479. doi: 10.3389/fpsyt.2020.585479, PMID: 33510656 PMC7835428

[23] Edward K Castle D Mills C Davis L Casey J. An integrative review of paternal depression. Am J Mens Health. (2015) 9:26–34. doi: 10.1177/1557988314526614, PMID: 24626601

[24] Kennedy E Munyan K. Sensitivity and reliability of screening measures for paternal postpartum depression: an integrative review. J Perinatol. (2021) 41:2713–21. doi: 10.1038/s41372-021-01265-6, PMID: 34974537 PMC8752439

[25] Shafian AK Mohamed S Nasution Raduan NJ Hway Ann AY. A systematic review and meta-analysis of studies validating Edinburgh Postnatal Depression Scale in fathers. Heliyon. (2022) 8:e09441. doi: 10.1016/j.heliyon.2022.e09441 35663736 PMC9156997

[26] Cox JL Holdenand JM Sagovsky R. Detection of postnatal depression development of the 10-item Edinburgh Postnatal Depression Scale. Br J Psychiatry. (1987) 150:782–6. doi: 10.1192/bjp.150.6.782 3651732

[27] Beck AT Ward CH Mendelson M Mock J Erbaugh J. An inventory for measuring depression. Arch Gen Psychiatry. (1961) 4:561–71. doi: 10.1001/ARCHPSYC.1961.01710120031004 13688369

[28] Radloff LS. The CES-D scale: a self-report depression scale for research in the general population. Appl Psychol Meas. (1977) 1:385–401. doi: 10.1177/014662167700100306

[29] Whittemore R Knafl K. Prenatal and postpartum depression in fathers and its association with maternal depression a meta-analysis. JAMA. (2005) 52:546–53. doi: 10.1111/j.1365-2648.2005.03621.x 20483973

[30] Kroenke K Spitzer RL Williams JBW. The PHQ-9 validity of a brief depression severity measure. J Gen Intern Med. (2001) 16:606–13. doi: 10.1046/j.1525-1497.2001.016009606.x 11556941 PMC1495268

[31] Tran TD Tran T Fisher J. Validation of three psychometric instruments for screening for perinatal common mental disorders in men in the north of Vietnam. J Affect Disord. (2011) 136:104–9. doi: 10.1016/j.jad.2011.08.012, PMID: 21907417

[32] Baldoni F Giannotti M. Perinatal distress in fathers: toward a gender-based screening of paternal perinatal depressive and affective disorders. Front Psychol. (2020) 11:1892. doi: 10.3389/fpsyg.2020.01892, PMID: 32973604 PMC7461929

[33] Walsh TB Davis RN Garfield C. A call to action: screening fathers for perinatal depression. Pediatrics. (2020) 145:e20191193. doi: 10.1542/PEDS.2019-1193, PMID: 31879278

[34] Addis ME. Gender and depression in men. Clin Psychol Sci Pract. (2008) 15:153–68. doi: 10.1111/J.1468-2850.2008.00125.X

[35] Salokangas RKR Vaahtera K Pacriev S Sohlman B Lehtinen V. Gender differences in depressive symptoms an artefact caused by measurement instruments? J Affect Disord. (2002) 68:215–20. doi: 10.1016/S0165-0327(00)00315-3 12063149

[36] Gressier F Glangeaud-Freudenthal NMC Essadek A Falissard B Corruble E Sutter-Dallay AL. Impact of paternal psychiatric disorders on parents-baby separation after mother-baby unit care. Child Abuse Negl. (2024) 149:106652. doi: 10.1016/j.chiabu.2024.106652, PMID: 38277874

[37] Goodman JH. Paternal postpartum depression, its relationship to maternal postpartum depression, and implications for family health. J Adv Nurs. (2004) 45:26–35. doi: 10.1046/j.1365-2648.2003.02857.x, PMID: 14675298

[38] Philpott LF Phn P En P Hp P Corcoran P. Paternal postnatal depression in Ireland: prevalence and associated factors. Midwifery. (2017) 56:121–7. doi: 10.1016/j.midw.2017.10.009 29096280

[39] Ansari NS Shah J Dennis CL Shah PS. Risk factors for postpartum depressive symptoms among fathers: a systematic review and meta-analysis. Acta Obstet Gynecol Scand. (2021) 100:1186–99. doi: 10.1111/aogs.14109, PMID: 33539548

[40] Hambidge S Cowell A Arden-Close E Mayers A. “What kind of man gets depressed after having a baby?” fathers’ experiences of mental health during the perinatal period. BMC Pregnancy Childbirth. (2021) 21:463. doi: 10.1186/s12884-021-03947-7, PMID: 34187395 PMC8244226

[41] Fletcher R Dowse E St George J Payling T. Mental health screening of fathers attending early parenting services in Australia. J Child Health Care. (2017) 21:498–508. doi: 10.1177/1367493517732166, PMID: 29110526

